# Genome-Wide Analyses of the Soybean *GmABCB* Gene Family in Response to Salt Stress

**DOI:** 10.3390/genes16020233

**Published:** 2025-02-19

**Authors:** Hui Zou, Caiyun Fan, Xiulin Chen, Ruifeng Chen, Zhihui Sun, Xiaorong Wan

**Affiliations:** 1 Guangzhou Key Laboratory for Research and Development of Crop Germplasm Resources, Zhongkai University of Agriculture and Engineering, Guangzhou 510225, China; zzzouhui_11@163.com; 2 Guangdong Provincial Key Laboratory of Plant Adaptation and Molecular Design, Innovative Center of Molecular Genetics and Evolution, School of Life Sciences, Guangzhou University, Guangzhou 510642, China; 2112314022@e.gzhu.edu.cn (C.F.); 18022946230@163.com (X.C.); 17881961278@163.com (R.C.)

**Keywords:** soybean, ATP-binding cassette transporter family, ABCB, salt stress

## Abstract

Background: Soybean (*Glycine max* (L.) *Merr*.) is a significant economic oilseed crop, and saline-alkali soils restrict soybean yield. Identifying salt-tolerant genes is a key strategy for enhancing soybean productivity under saline-alkali conditions. The roles of the *GmABCB* gene family in growth, development, and stress resistance remain incompletely understood. Methods: Bioinformatics approaches were employed to identify and analyze *GmABCB* genes. A total of 39 *GmABCB* genes were identified and analyzed in the soybean genome, focusing on their phylogenetic relationships, chromosomal distribution, gene structure, cis-acting elements, evolutionary history, and expression patterns under salt stress. Results: A total of 39 *GmABCB* genes were identified. These genes are unevenly distributed across the soybean genome, with 21 segmental duplication events identified. Several cis-acting elements associated with abiotic stress responses were identified. Conclusions: The *GmABCB* gene family likely regulates growth, development, and stress tolerance in soybean.

## 1. Introduction

Soybean is a vital economic oilseed crop that serves as a critical source of plant-based protein and oil for both human and animal nutrition [1]. China is the world’s largest soybean consumer; however, the domestic average yield remains low, resulting in a self-sufficiency rate of less than 15%, with over 85% of its soybean supply reliant on imports. Soil salinization is a major constraint on agricultural productivity, affecting approximately 950 million hectares of saline–alkali land worldwide, with nearly 25% of arable land (about 57 million hectares) impacted to varying degrees [2,3]. Of the 230 million hectares of irrigated land globally, an estimated 20–30% is affected by soil salinization to some degree [4]. In soybean, numerous QTLs related to salt tolerance have been identified, with the gene encoding the cation/H^+^ antiporter *GmSALT3* (*SALT TOLERANCE-ASSOCIATED 3*) serving as a key salt tolerance gene [5]. GmSALT3 is an endomembrane-localized protein with transport activity that facilitates the exclusion of Na^+^ and Cl^−^ from the aerial parts of the plant by restricting Na^+^ loading into the xylem. Simultaneously, Cl^−^ is re-translocated from the aerial parts to the roots through the phloem, promoting phloem-mediated Cl^−^ recirculation and improving salt tolerance [6,7]. Through genome-wide association studies (GWAS) and transcriptome sequencing (RNA-seq), the key protein GmNPF7.5 (PEPTIDE TRANSPORTER FAMILY) responsible for Cl^−^ transport was identified. Yeast two-hybrid screening identified GmPI4Kγ4 (PHOSPHATIDYLINOSITOL-4-KINASE) as an interacting partner of GmNPF7.5. The kinase GmPI4Kγ4 phosphorylates GmNPF7.5, specifically inhibiting its Cl^−^ transport activity without impacting its NO_3_^−^ transport efficiency. This action effectively reduces Cl^−^ accumulation in the plant, thereby significantly enhancing the salt tolerance of soybean [8]. Furthermore, salt stress-related genes have been progressively identified through reverse genetics, primarily focusing on transcription factors. For instance, under salt stress, *GmSIN1* (*SALT INDUCED NAC1*), *GmNCED3s* (*9-CIS-EPOXYCAROTENOID DIOXYGENASES*, key genes in ABA synthesis), and *GmRbohBs* (*RESPIRATORY BURST OXIDASE HOMOLOG B*, key enzyme genes in ROS synthesis) establish a positive feedback loop. This loop facilitates the rapid transformation and amplification of initial salt stress signals into ABA and ROS signaling pathways. Within a specific concentration range, these signals act synergistically to promote soybean root elongation and enhance salt tolerance [9]. Recent studies have shown that miR160a enhances salt tolerance in soybean by cleaving its target gene, *GmARF16* (*AUXIN RESPONSE FACTOR*). GmARF16 functions as an upstream regulator, directly activating the expression of *GmMYC2*. In turn, GmMYC2 negatively regulates proline synthesis, making soybean more susceptible to salt stress [10]. GmERF13 (ETHYLENE RESPONSIVE FACTOR 13) interacts with the key nodulation factor GmLBD16a (LATERAL ORGAN BOUNDARIES DOMAIN 16), inhibiting its binding to the promoter of its downstream gene *GmEXP17c* (*EXPANSIN 17c*), thereby affecting the transcriptional activity of *GmLBD16a*. Additionally, *GmABI5* (*ABSCISIC ACID INSENSITIVE 5*) directly binds to the promoter of *GmERF13* to regulate its expression. These interactions highlight the critical roles of *GmERF13* and *GmABI5* in suppressing nodule formation and nitrogen fixation in soybean under salt stress [11]. Consequently, the identification of salt-tolerance genes and the accelerated breeding of salt-tolerant soybean cultivars represent essential strategies to enhance soybean productivity under saline conditions.

Adenosine triphosphate (ATP)-binding cassette (ABC) transporters play a crucial role in crop yield, quality development, and stress response mechanisms [12,13]. This large family is classified into eight subfamilies, such as *ABCA*, *ABCB*, and *ABCC*, according to homology, phylogenetic relationships, and domain organization defined by the HUGO system [14,15]. These proteins are ubiquitous across prokaryotic and eukaryotic organisms [16]. Many plant ABC transporters participate in translocating plant secondary metabolites [17]. The transporter activity of these proteins is conserved [18], primarily depending on ATP hydrolysis to power the movement of substrates against concentration gradients across membranes [19,20]. As the second-largest subfamily within the ABC family [21], ABCB transporters are predominantly localized to the plasma membrane, with a minority found in mitochondrial or chloroplast membranes [22]. ABCB family proteins feature a transmembrane domain (TMD) for substrate recognition and a nucleotide-binding domain (NBD) for ATP binding and hydrolysis [23,24,25]. They are classified into two categories based on domain count: half transporters, comprising one TMD and one NBD, and full transporters, containing two TMDs and two NBDs, which form two TMD-NBD units connected by a flexible domain of approximately 60 amino acids [25,26,27]. The presence of these specialized domains enhances the transport capacity of ABCB proteins, increasing their binding affinity for diverse compounds [28].

Plant stress responses are closely linked to a variety of hormones, such as abscisic acid (ABA), auxin, brassinosteroids (BRs), cytokinins (CK), ethylene (ET), gibberellic acid (GA), jasmonic acid (JA), salicylic acid (SA), and strigolactones (SL) [29,30]. Among these hormones, ABCB transporters are primarily responsible for the polar transport of auxin. The polar transport of auxin predominantly depends on the ABCB/PGP (ATP-BINDING CASSETTE B/P-GLYCOPROTEIN), PIN (PIN-FORMED), and AUX1/LAX (AUXIN1/LIKE AUXIN) transporter families, with ABCB playing a pivotal role in auxin transport in apical tissues and long-distance movement [31]. In *Arabidopsis*, not all ABCB proteins participate in auxin transport. Among the 22 full-size ABCBs, which contain two transmembrane domains and two nucleotide-binding domains [32], ABCB1, ABCB4, ABCB6, ABCB19, ABCB20, and ABCB21 are involved in auxin transport [33,34,35]. Among them, AtABCB1, located in the root, and AtABCB19, localized to the plasma membrane, both participate in the efflux of auxin [36,37]. AtABCB4/AtPGP4 (ATP-BINDING CASSETTE B4/P-GLYCOPROTEIN4) is reported to regulate the influx of auxin in root epidermal cells, thereby mediating the development of lateral roots and root hairs [38,39]. The functions of AtABCB6 and AtABCB20 in regulating auxin transport are redundant [35]. AtABCB21 maintains auxin levels in the pericycle to sustain transport at the leaf tip, and the loss of AtABCB21 reduces auxin transport to the root, delaying lateral root development [40]. Additionally, ABCB transporters modulate processes like plant tropisms and heavy metal resistance [41]. For instance, the ABCB half-transporter ATM3/AtABCB24 responds to cadmium and lead induction, and the increased sensitivity of the *atm3-1* (*sta1*) mutant to cadmium suggests that the ATM3 transporter is involved in heavy metal tolerance [42]. ALS1/TAP2 (AtABCB27) is reported to be involved in aluminum transport, and the roots of the *als1-1* mutant exhibit increased sensitivity to aluminum [43]. Members of the *ABCB* gene family also play crucial roles in other crops. In maize, the *ZmABCB15* gene encodes a polar auxin transporter, and polar auxin transport may contribute to resistance against RBSDV (*Rice black-streaked dwarf virus*) by influencing the distribution of endogenous auxin in tissues. Overexpression of *ZmABCB15* can enhance resistance by reducing the replication rate of RBSDV [44]. Maize *BR2* (*BRACHYTIC 2*), homologous to the *Arabidopsis* *AtABCB1* gene, is involved in auxin transport. Mutations in *BR2* reduce basipetal auxin transport, resulting in dwarfism, a phenotype resembling that of the *Arabidopsis* *atabcb1* mutant. This suggests that ABCB proteins may similarly regulate auxin transport in monocots [45]. Additionally, *ZmABCB7* and *ZmABCB8* are significantly induced by drought stress, whereas *ZmABCB18* is notably repressed by salt stress [46]. In rice, OsABCB14 is localized to the plasma membrane, and its knockout results in reduced auxin concentration and polar auxin transport rates. Furthermore, osabcb14 mutants were found to be insensitive to iron deficiency treatment [47]. Other members, such as *OsABCB11*, *OsABCB8*, *OsABCB13*, *OsABCB23*, and *OsABCB24*, are induced by drought stress, whereas *OsABCB6*, *OsABCB9*, and *OsABCB8* are induced by salt stress [48,49]. These findings highlight the diverse roles of ABCB transporters in various aspects of plant life processes.

Recently, ABCB subfamily proteins have been implicated in abiotic stress responses in monocotyledonous plants, while their roles in soybean remain unclear. This study utilizes bioinformatics to perform a comprehensive genome-wide analysis of the soybean *GmABCB* gene family, examining their functions and responses. A total of 39 *GmABCB* genes were identified, and their phylogenetic relationships, chromosomal distribution, gene structure, cis-acting elements, evolutionary history, and expression patterns under salt stress were analyzed at the soybean genome level. The results of this study serve as a reference for advancing the understanding of soybean *GmABCB* functions and evolution, facilitating the identification of salt-tolerant genes within this gene family.

## 2. Materials and Methods

### 2.1. Plant Materials, Growth Conditions, and Treatment Methods

The soybean variety Williams 82 (Wm 82) was used for salt stress experiments. This variety is sourced from the Guangdong Provincial Key Laboratory of Plant Adaptation and Molecular Design, Innovation Center for Molecular Genetics and Evolution. The seedlings were cultivated in a greenhouse at 25 °C with photoperiod 16 h light/8 h dark and 70% relative humidity. Seed of uniform size were selected and germinated in vermiculite until the first true leaves fully expanded, after which they were transferred to a half-strength Hoagland nutrient solution with a pH of 6.0 for hydroponic cultivation. The saline treatment method was described previously [6,50,51]. When the V1 growth stage (the first trifoliate growth stage) was achieved, the seedlings were removed from the pots and held with the foam floats in the 0 mM (Mock) or 200 mM NaCl solution for the saline treatment. Samplings were focused on the leaf and root tissue after 0, 6, 12, and 24 h (h) after the abiotic stress treatments. Collected samples were immediately wrapped in aluminum foil, flash-frozen in liquid nitrogen, and stored at −80 °C for subsequent RNA extraction.

### 2.2. Database Search for GmABCB Genes in Soybean

In order to identify the members of the *GmABCB* genes, the amino acid sequences of the *GmABCB* genes family were utilized for BLAST 2.16.0 comparison with those of *Arabidopsis thaliana* via the Phytozome (https://phytozome-next.jgi.doe.gov/, accessed on 15 June 2024) and NCBI databases (https://www.ncbi.nlm.nih.gov, accessed on 15 June 2024). Protein domain validation of the candidate gene members was performed using the NCBI Conserved Domain Database (CDD) (https://www.ncbi.nlm.nih.gov/cdd/, accessed on 16 June 2024) and SMART (http://smart.embl.de/, accessed on 16 June 2024). The biochemical properties of the *GmABCB* genes, including sequence length, molecular weight, isoelectric point, and size, were analyzed using TBtools software.v2.097 The subcellular localization of GmABCB proteins was predicted using the WoLF PSORT online tool (https://wolfpsort.hgc.jp/, accessed on 20 June 2024).

### 2.3. Phylogenetic Analysis, Chromosomal Distribution, and Gene Duplication Analyses

The GmABCB and AtABCB protein sequences were employed for multiple sequence alignment using the MEGA7 software and the Clustal W algorithm. A phylogenetic tree was generated from the alignment results using the maximum likelihood (ML) method with 1000 bootstrap replicates. All gene names are listed in Table S1. The chromosomal distribution of *GmABCB* genes was visualized by importing genome and gene position files into TBtools. Gene duplication events of *GmABCB* genes were analyzed using the one-step method in the MCScanX plugin of TBtools software v2.097.

### 2.4. Gene Structure and Promoter Cis-Acting Elements Analyses

To better understand the gene structure of the ABCB family, CDS and genomic data were obtained from Phytozome. Use the GSDS online tool (http://gsds.gao-lab.org/, accessed on 22 June 2024) to generate exon–intron structure diagrams. Identify conserved motifs with the MEME online tool (https://meme-suite.org/meme/tools/meme, accessed on 22 June 2024), and visualize and annotate these motifs using TBtools software v2.097. Additionally, cis-acting elements in the 2000 bp promoter regions upstream of the 39 *GmABCB* genes in the soybean genome were predicted using the Plant CARE database.

### 2.5. RNA Isolation and qRT-PCR Analysis

Total RNA was extracted using the Ultrapure RNA Kit according to the manufacturer’s protocols. The RNA quality was evaluated using the Agilent 2100 Bioanalyzer (Agilent Technologies, Palo Alto, CA, USA) and further confirmed through RNase-free agarose gel electrophoresis. cDNA synthesis was performed using 500 μg of RNA with the PrimeScript RT Reagent Kit, which includes a gDNA Eraser (Takara, Japan), with Gmactin serving as the reference gene. Quantitative PCR (qPCR) analysis was conducted utilizing the ChamQ Universal SYBR qPCR Master Mix, following the manufacturer’s guidelines. Three independent experiments were carried out, all yielding consistent results. Statistical analysis was conducted through One-way Analysis of Variance (ANOVA), and Duncan’s multiple range test was employed (*p* < 0.05, n = 3). The NCBI (https://www.ncbi.nlm.nih.gov, accessed on 10 July 2024) was used to design gene-specific quantitative primers for *GmABCBs*, and amplification specificity was validated by agarose gel electrophoresis and melt-curve analysis. The primers used for the qRT-PCR analysis are detailed in Table S2.

## 3. Results

### 3.1. Identification of GmABCB Genes in Soybean

**Figure 1:**
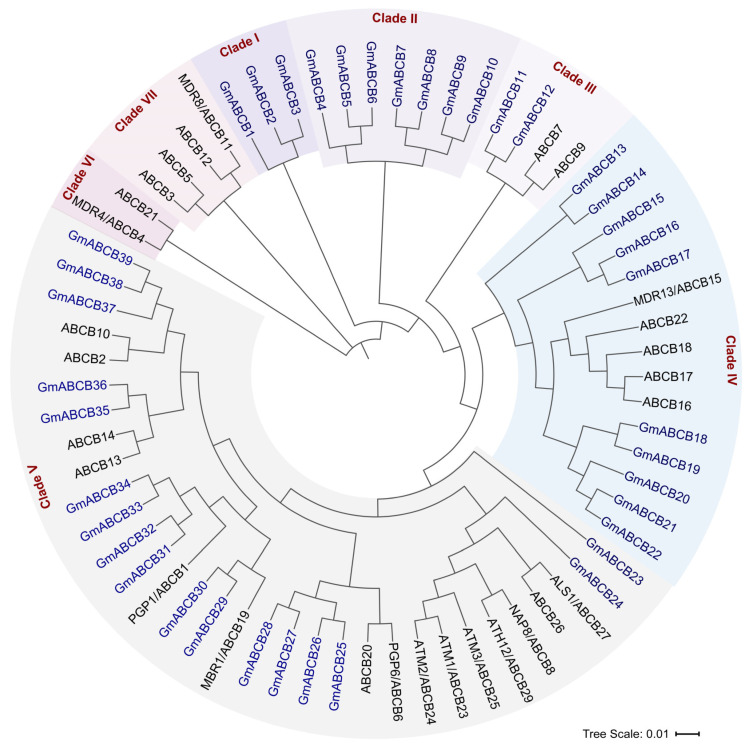
Phylogenetic tree of the ABCB family proteins in soybean and *Arabidopsis* constructed using the neighbor-joining method. The phylogenetic tree was constructed using MEGA 7.0, employing the maximum likelihood method with 1000 bootstrap replicates. The tree is divided into seven clades (I–VII), each represented by a distinct color. The 28 AtABCB proteins from *Arabidopsis* are labeled in black, while the 39 GmABCB proteins obtained from soybean are labeled in purple-blue.

Candidate GmABCB family proteins encoded in the soybean genome were identified through an extensive BLAST search using known *Arabidopsis AtABCB* genes as references. The accuracy of the protein predictions was subsequently validated using NCBI CDD (https://www.ncbi.nlm.nih.gov/cdd/, accessed on 16 June 2024) and SMART (http://smart.embl.de/, accessed on 16 June 2024). In total, 39 GmABCB proteins were identified (Supplementary Tables S1 and S6). Then, we clustered GmABCB proteins clustered with 28 AtABCB protein sequences from *Arabidopsis*. Based on the phylogenetic tree, GmABCB proteins were named sequentially from GmABCB1 to GmABCB39 according to their proximity to corresponding *Arabidopsis* branches, The ABCB family members were subsequently grouped into seven distinct clades, designated Clade I through Clade VII. Of these clades, Clade V contained the largest number of members (17), followed by Clade IV (10), Clade II (7), and Clade I (3). Clade III contained two members, whereas Clade6 and Clade7 had no GmABCB family members within the soybean family (Figure 1; Supplementary Table S1). Amino acid sequence analysis of the GmABCB family revealed protein lengths ranging from 1237 amino acids (GmABCB13) to 1515 amino acids (GmABCB23), with molecular weights ranging from 135.6 to 166.5 kDa, and predicted isoelectric points (pI) spanning from 6 to 8.93. Subcellular localization of GmABCB proteins was predicted using the WoLF PSORT tool (https://wolfpsort.hgc.jp/, accessed on 20 June 2024), revealing that 37 members are predicted to localize to the plasma membrane, whereas GmABCB7 and GmABCB8 are likely localized to the nucleus.

### 3.2. Analysis of the Chromosomal Localization of GmABCB Genes in Soybean

**Figure 2:**
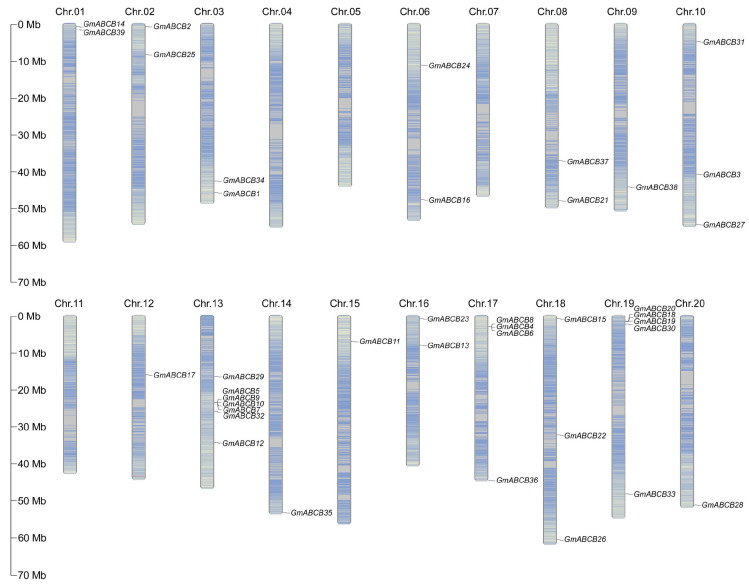
Chromosomal location analysis of *GmABCB* genes in the soybean genome. The chromosomes containing *GmABCB* genes are drawn to scale, with chromosome numbers displayed at the top of each bar, and distances measured in megabases (Mb). A scale bar indicates 10 Mb. Gene names are displayed to the right of each chromosome, corresponding to their respective locations.

The chromosomal locations of the *GmABCB* family genes were extracted from the soybean genome annotation file, and the 39 *GmABCB* family members were mapped onto the soybean genome (Figure 2). The results revealed an uneven distribution of *GmABCB* genes across the chromosomes. Chromosomes Chr04, Chr05, Chr07, and Chr11 lack any *GmABCB* genes, while all other chromosomes carry at least one member of the *GmABCB* family. Chr13 harbors the highest number of *GmABCB* genes, with seven members, followed by Chr19, which contains five members. Notably, no positive correlation was observed between chromosome length and the number of *GmABCB* genes present on each chromosome.

### 3.3. Gene Duplication Events and Collinearity Analysis of the GmABCB Genes

**Figure 3:**
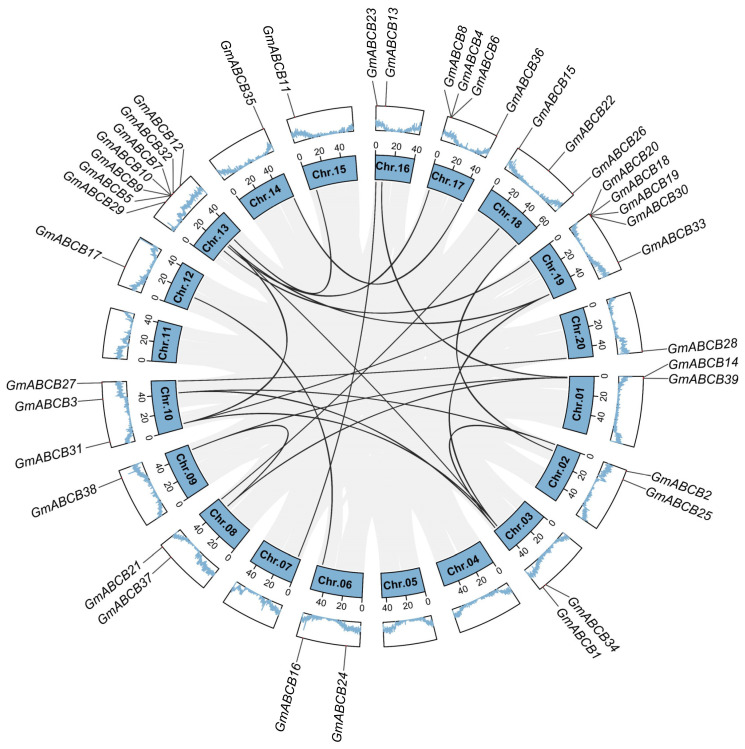
Gene duplication events and collinearity analysis of *GmABCB* genes. Chromosomal distribution and inter-chromosomal relationships of *GmABCB* genes. Black curves represent duplicated *GmABCB* gene pairs. The outer circle displays the positions of these genes, with trapezoidal boxes representing different chromosomes (Chr1–Chr20). Blue zigzag lines illustrate the gene density across each chromosome.

Gene duplication is a prevalent and essential phenomenon in plant evolution. The evolution of gene families is predominantly influenced by whole-genome duplication, segmental duplication, and tandem duplication. In this study, no *GmABCB* genes were identified as tandem duplicates, whereas 21 pairs of segmental duplication events were detected (Figure 3). The presence of collinear genes highlights both the similarities and differences in plant evolutionary processes and underscores the conservation of genomic structures. Collinear genes are orthologous genes conserved in species order; however, speciation can lead to changes in the number and positions of these genes. Consequently, collinearity analysis provides insights into the evolutionary relationships between species. Comparative genomic collinearity maps were constructed using cultivated soybean W82, wild soybean, and ZH13 (Figure S1, Supplementary Table S5) to investigate the evolutionary relationships of *GmABCB* genes among soybean species. The findings indicate that *GmABCB* genes exhibit a closer phylogenetic and evolutionary relationship with ZH13.

### 3.4. Structural Analysis of GmABCB Proteins

**Figure 4:**
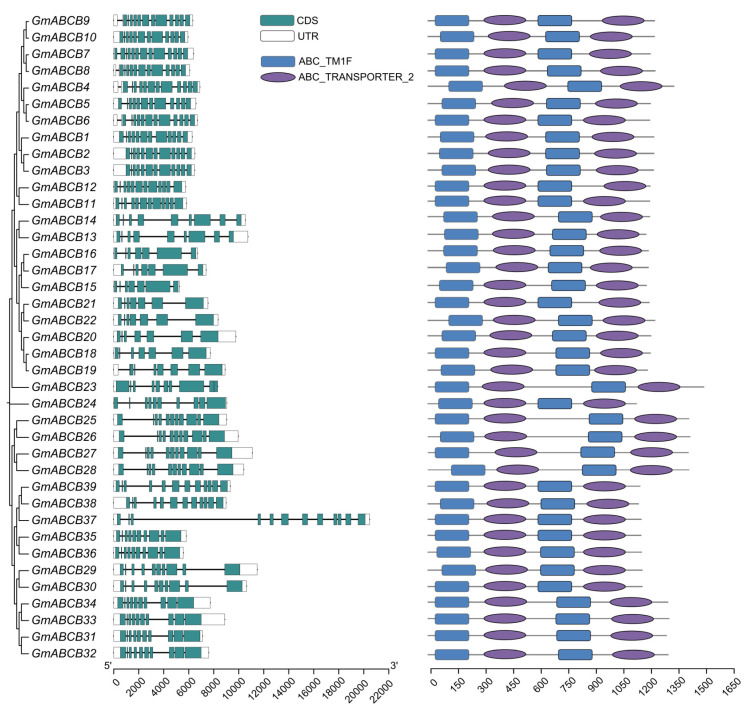
Structural analysis of GmABCB Proteins. White boxes represent the untranslated 5′ and 3′ regions; turquoise boxes indicate exons; black lines indicate introns. The diverse conserved domains were represented with different-colored boxes. ABC_TM1F represents the ABC transporter integral membrane type-1 fused domain; ABC_TRANSPORTER_2 represents the ATP-binding cassette, ABC transporter-type domain. In addition, the length of the gene structure is estimated using the black scale lines at the bottom of the figure.

To further investigate the evolutionary conservation of the *GmABCB* gene family, the gene structures were analyzed (Figure 4). The exons and introns of the 39 *GmABCB* genes were predicted based on their coding sequences and genomic information. The analysis revealed that the number of exons in the *GmABCB* genes ranged from six to thirteen. Consistent with the structural characteristics of GmABCB proteins, all 39 GmABCB family members were identified as full-size transporters containing the ABC_TM1F domain (representing the ABC transporter transmembrane type 1 fusion domain) and the ABC_TRANSPORTER_2 domain (representing the ATP-binding cassette).

### 3.5. Promoter Cis-Acting Elements of the GmABCB Genes

**Figure 5:**
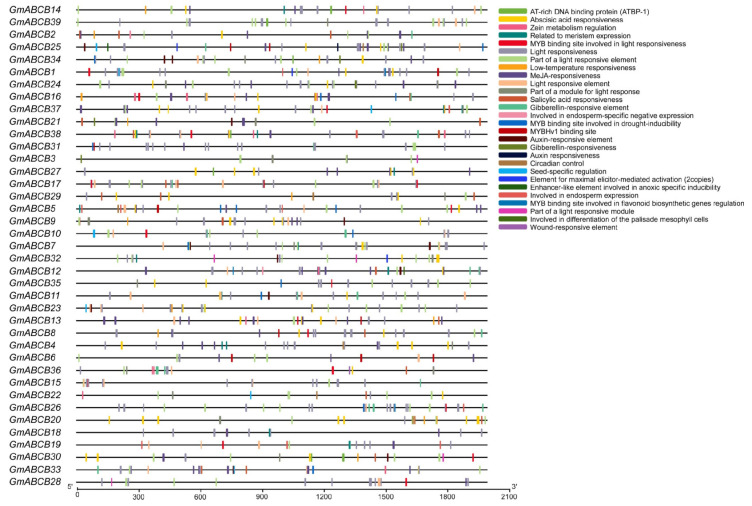
Promoter cis-acting elements of *GmABCB* genes. This figure illustrates the cis-acting elements identified in the promoter regions of the *GmABCB* genes family members. A total of 28 distinct cis-regulatory elements were identified in the promoter regions of the *ABCB* genes, each represented by a unique color in the legend on the right. The promoter sequence lengths are indicated by the scale bar at the bottom of the figure.

To investigate the regulatory mechanisms of *GmABCB* genes, the PlantCARE database was used to predict cis-acting elements within the 2000 bp promoter regions upstream of the *GmABCB* genes in the soybean genome. A total of 28 cis-regulatory elements were identified in the promoter regions of the *GmABCB* genes (Figure 5), including hormone-related elements such as abscisic acid response elements, jasmonic acid response elements, gibberellin response elements, and auxin response elements. These findings suggest that the expression of *GmABCB* genes may be regulated by plant hormones. Some *GmABCB* genes harbor elements associated with environmental stress responses, including MYB binding sites, which are involved in drought inducibility, low-temperature responsiveness, and enhancer-like elements linked to anoxic-specific inducibility. Additionally, the promoters include numerous light-responsive elements, growth and development-related elements, metabolic regulation elements, as well as circadian rhythm and defense-related elements. These results indicate that variations in cis-acting elements contribute to temporal and spatial differences in the expression of *GmABCB* gene family members, as well as their diverse responses to environmental stimuli.

### 3.6. Expression Patterns of GmABCB Genes in Different Tissues

**Figure 6:**
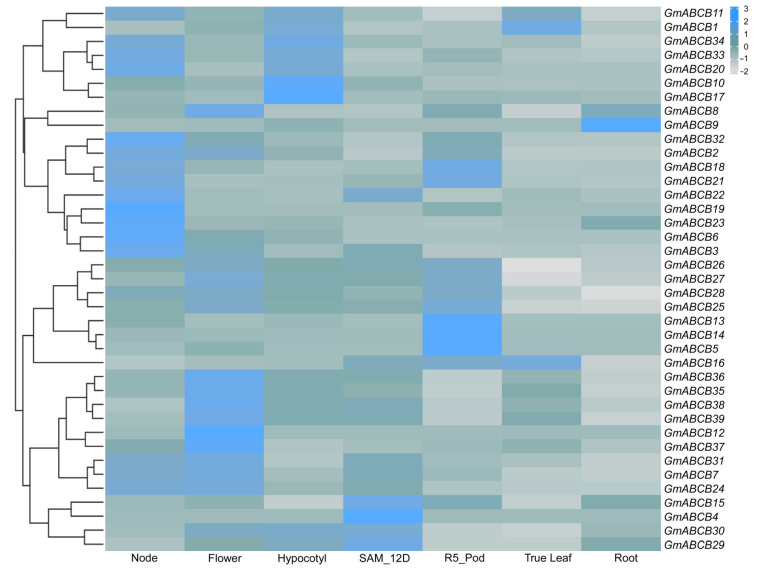
Expression heatmap of the *GmABCB* gene family in seven soybean tissues. The heatmap shows the expression levels of the *GmABCB* gene family in seven soybean tissues: root nodules, flowers, hypocotyls, shoot apical meristems, pods, leaves, and roots. FPKM (Fragments Per Kilobase of transcript per Million mapped reads) values were retrieved from the Phytozome online dataset. Colors in the heatmap represent FPKM values, as indicated by the color scale in the upper right corner.

To investigate the expression patterns of *GmABCB* genes across developmental stages and tissue types, FPKM data from the Soybean Phytozome database (https://phytozome-next.jgi.doe.gov/, accessed on 20 July 2024) were utilized for computational analysis of the expression levels of *GmABCB* genes in soybean root nodules, flowers, hypocotyls, shoot apical meristems (SAM), pods, leaves, and roots (Supplementary Table S3). The results, as depicted in Figure 6, reveal that most *GmABCB* genes exhibit high expression levels in root nodules, flowers, hypocotyls, and pods. Conversely, *GmABCB4*, *GmABCB15*, *GmABCB22*, and *GmABCB29* display relatively higher expression levels in SAM, while *GmABCB1*, *GmABCB11*, and *GmABCB16* show elevated expression in leaves compared to other genes. Certain *GmABCB* genes demonstrate tissue-specific expression, including *GmABCB9* in roots, *GmABCB4* in SAM, *GmABCB12* in flowers, and *GmABCB14* in pods. The differential expression patterns of *GmABCB* genes across soybean tissues suggest that the *GmABCB* gene family plays diverse roles in regulating soybean growth and development.

### 3.7. Quantitative Expression Profiles of GmABCB Genes in Root and Leaves Under Salt Stress

**Figure 7:**
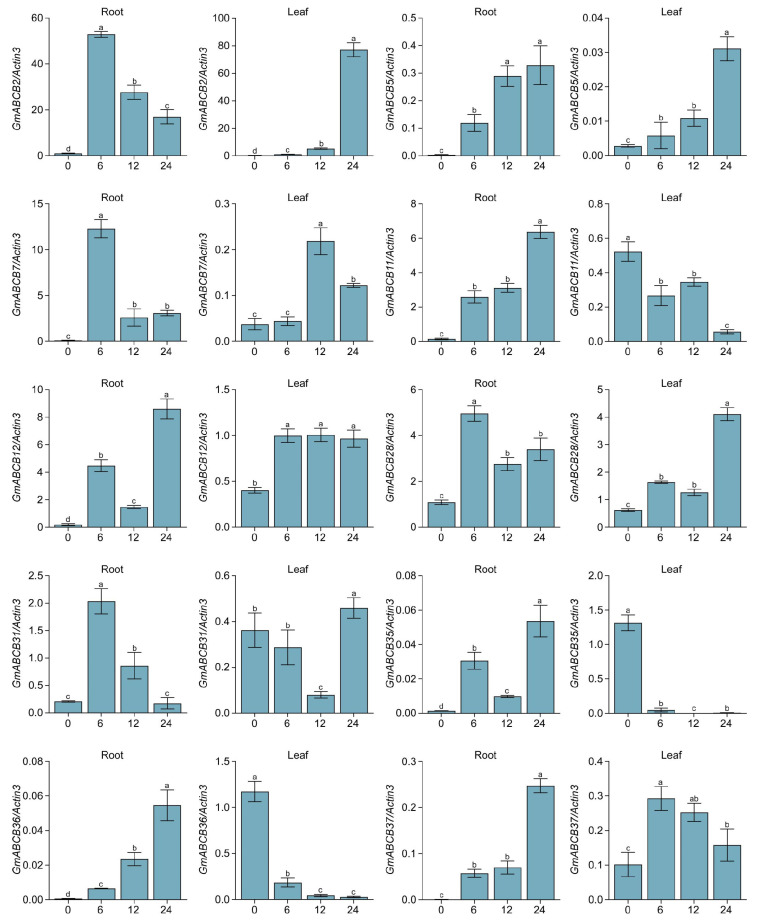
The qPCR results of 10 *GmABCB* genes in roots and leaves under salt stress. Data were normalized to the *Actin3* gene, and vertical bars indicate the standard deviations. Statistical analysis was conducted through One-way Analysis of Variance (ANOVA), and Duncan’s multiple range test was employed (*p* < 0.05, n = 3). Significant differences were indicated by different letters.

Root systems are frequently subjected to diverse abiotic stresses and act as the primary tissue responding to these challenges. Using RNA-seq data generated in our laboratory, we analyzed the expression patterns of *GmABCB* genes in soybean roots and leaves under untreated and salt-stressed conditions (Supplementary Table S4). Under salt stress, the expression of *GmABCB35*, *GmABCB36*, and *GmABCB37* was significantly upregulated in leaves. Similarly, the expression of *GmABCB2*, *GmABCB5*, *GmABCB7*, *GmABCB11*, *GmABCB12*, *GmABCB28*, and *GmABCB31* was significantly upregulated in roots following salt treatment (Figure S2). Consequently, qRT-PCR was employed to examine the transcriptional expression patterns of the 10 salt stress-responsive genes in roots and leaves at 0, 6, 12, and 24 h following 200 mM NaCl treatment (Figure 7). Among these genes, *GmABCB2* expression was significantly upregulated in both roots and leaves under salt treatment, with the most rapid response detected in roots. These findings further support the hypothesis that *GmABCB2* plays a critical role in the salt stress response pathway, warranting further in-depth investigation.

## 4. Discussion

ABCB transporters participate in multiple physiological processes in plants, such as polar auxin transport, phototropism, gravitropism, organogenesis, and heavy metal transport. In 1992, *AtPGP1/AtABCB1*, the first gene to be identified within the ABCB subfamily was cloned from *Arabidopsis* [52]. Recently, *Arabidopsis* ABCB19 was identified as the first brassinosteroid (BR) transmembrane transport protein in plants. This discovery provides a systematic understanding of the molecular process through which ABCB19 recognizes and transports brassinolide, addressing a critical gap in BR signaling pathway research and clarifying the functional mechanism of this protein [53]. Genome-wide expression analysis in rice revealed that *OsABCB10* is highly expressed in anthers and pollen, indicating its crucial role in their development [54]. Expression profiling under abiotic stress demonstrates altered expression levels for most *ABCB* genes. For example, *OsABCB8*, *OsABCB11*, *OsABCB13*, *OsABCB23*, and *OsABCB24* are upregulated by drought stress, whereas *OsABCB6*, *OsABCB9*, and *OsABCB8* are upregulated by salt stress. Nevertheless, the functional roles of *OsABCB* subfamily genes in rice responses to abiotic stress demand further experimental validation [50,51].

Soybean is classified as a moderately salt-sensitive crop, with yields potentially decreasing by up to 40% under elevated salinity levels [2]. Multiple salt tolerance-related QTLs have been identified in soybean, with *GmSALT3* (which is salt tolerance-associated), which encodes a cation/H^+^ transporter, recognized as a key gene for salt tolerance. This finding suggests that cation/H^+^ transporter proteins are under selective pressure related to salt tolerance during plant evolution and breeding programs [5,6,7]. Consequently, enhancing soybean salt tolerance, developing salt-tolerant soybean varieties, and identifying key components of the plant salt tolerance network remain critical objectives. The ABCB family, a large multigene family in plants, plays a pivotal role in responding to adverse environmental conditions during plant growth and development. Although members of the ABCB family have been implicated in abiotic stress responses in rice, corn, and sorghum [46,55,56], their role in abiotic stress responses in soybean remains unexplored. In this study, 39 members of the *GmABCB* gene family in soybean were identified through whole-genome analysis, followed by a phylogenetic analysis of these genes. These genes were grouped into seven distinct clades (Clade I–VII), with Clade V comprising the largest number of members (17), whereas Clade III contained only 2 members. Compared to the *ABCB* gene family in *Arabidopsis thaliana*, the *GmABCB* gene family in soybean shows greater diversity, potentially due to the high genomic complexity and polyploidization events that occur in soybean [57]. Phylogenetic analysis indicated that *GmABCB* genes exhibit high homology with *AtABCB* genes in *Arabidopsis*, particularly in Clade I and Clade II, suggesting that these genes may have conserved similar functions throughout evolution.

The *GmABCB* genes are unevenly distributed across the soybean genome, with higher concentrations observed for Chr13 and Chr19, whereas no *GmABCB* genes are present on Chr04, Chr05, Chr07, and Chr11. This non-uniform distribution may be linked to localized gene duplication events in the soybean genome. In this study, 21 segmental duplication events were identified within the *GmABCB* gene family, whereas no tandem duplication events were detected. Segmental duplication is a prevalent phenomenon in plant genome evolution and is frequently associated with whole-genome duplication events [58]. These duplication events may drive functional diversification of genes, thereby enhancing the plant’s adaptability to environmental stresses. Additionally, a comparative analysis of genome collinearity among cultivated soybean W82, wild soybean, and ZH13 revealed that the *GmABCB* genes exhibit a high degree of evolutionary conservation. Notably, the *GmABCB* genes exhibit a closer evolutionary relationship with ZH13, suggesting that these genes may have undergone selective pressure during soybean domestication, thereby preserving functions associated with stress response.

Structural analysis of the *GmABCB* genes revealed that all members contain the conserved ABC_TM1F and ABC_TRANSPORTER_2 domains. This structural conservation further corroborates the role of *GmABCB* genes in transmembrane transport. Additionally, an analysis of cis-acting elements in the promoter regions indicated that *GmABCB* genes are likely regulated by multiple hormones and environmental stimuli. For instance, the promoter regions of numerous *GmABCB* genes harbor response elements for abscisic acid (ABA), jasmonic acid (JA), and auxin, which are pivotal in plant responses to abiotic stresses such as salinity and drought [59]. In recent years, strigolactones (SLs) have been recognized as a novel class of plant hormones with crucial roles in regulating root architecture, symbiotic mycorrhizal formation, and stress responses. Studies have demonstrated that SLs not only regulate plant growth and development but also play a critical role in plants’ responses to abiotic stresses, including salinity [30]. SLs may interact with other hormones, such as auxin and ABA, to regulate plant stress responses through the modulation of hormone transport and signaling pathways. Therefore, investigating the interactions between SLs and the *ABCB* gene family in the context of hormone transport and stress responses may yield novel insights into the salt tolerance mechanisms of soybean.

Additionally, the promoter regions harbor MYB binding sites linked to low-temperature responsiveness, drought inducibility, and anoxia-specific inducibility. This implies that *GmABCB* genes may play a role in responding to various environmental stresses. The diversity of these cis-acting elements may explain the differential expression patterns of *GmABCB* genes across different tissues and under various stress conditions.

Analysis of the expression patterns of *GmABCB* genes across different tissues revealed that most *GmABCB* genes are highly expressed in root nodules, flowers, hypocotyls, and pods, while their expression is comparatively lower in leaves and roots. This tissue-specific expression pattern implies that *GmABCB* genes may play distinct roles during various developmental stages of soybean. For instance, *GmABCB4*, *GmABCB15*, and *GmABCB22* exhibit high expression in the shoot apical meristem (SAM), potentially reflecting their regulatory roles in apical growth and development.

Salt stress is a significant abiotic factor influencing soybean yield, yet the role of the *ABCB* gene family in plant responses to salt stress remains incompletely understood. Under salt stress conditions, the expression patterns of *GmABCB* genes exhibit marked alterations. Specifically, the expression of *GmABCB2*, *GmABCB5*, and *GmABCB7* is notably upregulated in roots, whereas the expression of *GmABCB35*, *GmABCB36*, and *GmABCB37* is markedly increased in leaves. These findings imply that these genes may contribute to enhanced plant salt tolerance by regulating ion transport, hormone signaling, and redox balance. Furthermore, the promoter regions of *GmABCB* genes harbor ABA-responsive elements, with ABA being a crucial hormone influencing plants’ responses to salt stress. ABA mitigates the detrimental effects of salt stress on plants by regulating stomatal closure and ion transport [60]. Consequently, *GmABCB* genes may participate in salt stress responses via the ABA signaling pathway. The rapid response of *GmABCB2* in both roots and leaves suggests that this gene may play a pivotal role in salt stress signaling, warranting further investigation.

Despite the comprehensive genomic analysis of the *GmABCB* gene family in soybean presented in this study, several questions have yet to be addressed. For example, the specific functional mechanisms of *GmABCB* genes under salt stress remain unclear, particularly regarding their roles in ion transport and hormone signaling. Moreover, functional validation of *GmABCB* genes using gene-editing technologies, such as CRISPR/Cas9, could provide insights into their precise roles in plant salt tolerance.

## 5. Conclusions

In this study, a comprehensive genome-wide analysis of the ATP-binding cassette (ABC) transporter family in soybean was carried out, with a particular focus on the ABCB subfamily, which plays a crucial role in crop yield, quality improvement, and stress responses. Through analyses of phylogenetic relationships, chromosomal distribution, gene structures, cis-acting elements, evolutionary history, and expression profiles under salt stress conditions, 39 *GmABCB* genes were identified and systematically characterized. The research findings indicated that the distribution of these genes on chromosomes is uneven, and specific *GmABCB* members may play important roles in salt stress responses. This study provides valuable insights into the functional roles and evolutionary dynamics of the *GmABCB* genes family in soybean, facilitating the identification of salt-tolerant genes and the breeding of salt-tolerant soybean cultivars.

## Data Availability

The data presented in this study are available in the Supplementary Materials.

## Supplementary Materials

The following supporting information can be downloaded at https://www.mdpi.com/article/10.3390/genes16020233/s1, Figure S1. Collinearity analysis of *ABCB* genes among W82, ZH13, and W05; Figure S2. Expression patterns of *GmABCB* genes under salt stress; Table S1. Physicochemical properties of *GmABCB* genes; Table S2. The primer sequences of *GmABCB* genes used for qRT-PCR; Table S3. FPKM values for soybean *GmABCB* genes in different tissues; Table S4. FPKM values of *GmABCB* genes in roots and leaves under salt stress; Table S5. List of ABCB genes; Table S6. Amino acid sequence of the *GmABCB* genes family.
