# Supplementary material for: Genome-Wide Analyses of the Soybean GmABCB Gene Family in Response to Salt Stress
Source: Genes (Basel). 2025 Feb 19;16(2):233. doi: 10.3390/genes16020233 (PMC11855854; doi:10.3390/genes16020233)
Supplement: Supplementary file 1 [file genes-16-00233-s001.zip › Supplementary Materials.pdf]

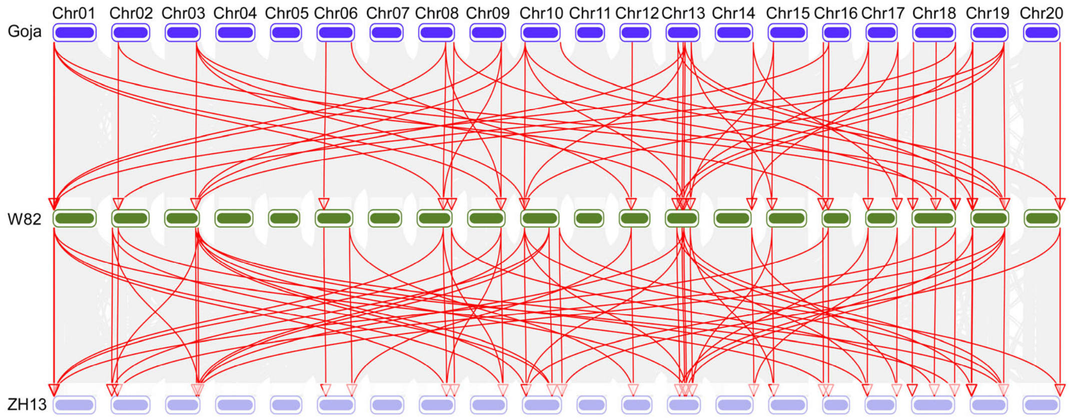

Figure S1. Collinearity analysis of *ABCB* genes among W82, ZH13, and W05. Gray lines represent extensive collinear blocks between cultivated soybean, wild soybean, and the ZH13 genome, while red lines indicate homologous relationships among *ABCB* genes. This figure illustrates the evolutionary relationships of the *ABCB* gene family among wild soybean PI483463, cultivated soybean W82 and ZH13 genome.

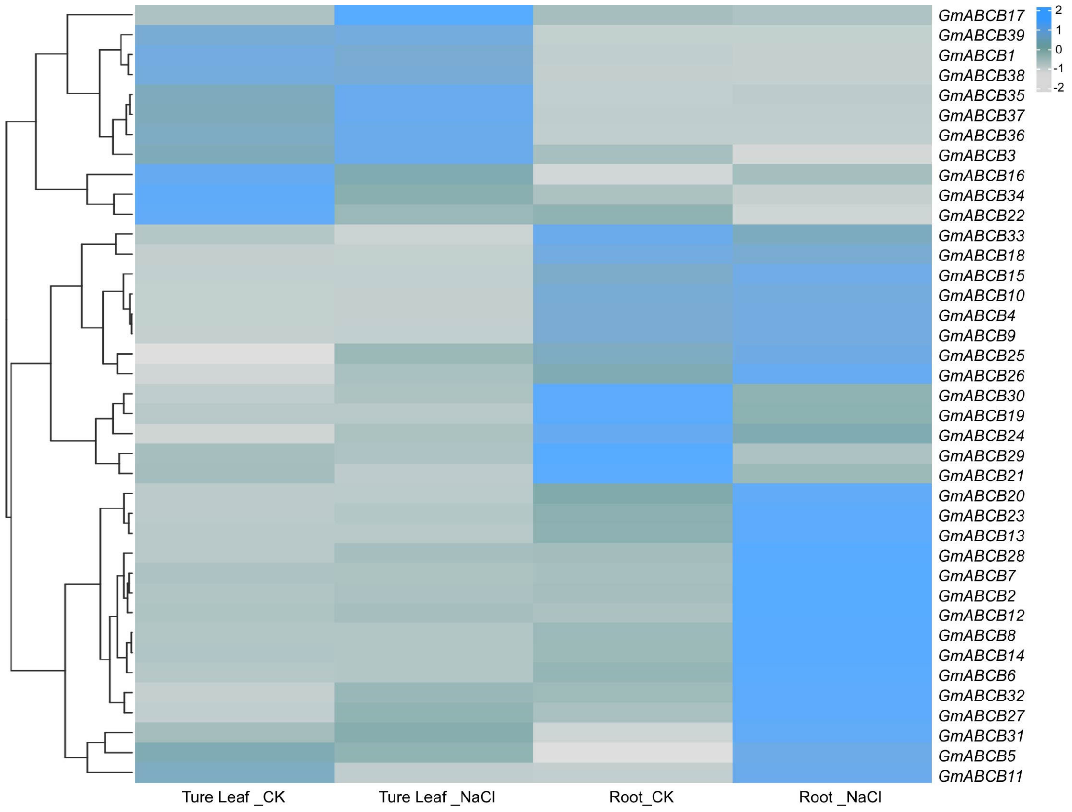

Figure S2. Expression patterns of *GmABCB* genes under salt stress. The heatmap is based on RNA-seq data, showing the expression levels of *GmABCB* genes in roots and leaves under salt treatment. Colors in the heatmap indicate expression levels, as shown by the color scale in the upper right corner.
